# Relief craving severity moderates nonpharmacological treatment outcomes in treatment‐seeking older adults with alcohol use disorder

**DOI:** 10.1111/acer.70097

**Published:** 2025-06-18

**Authors:** Peter Næsborg Schøler, Anette Søgaard Nielsen, Katie Witkiewitz, Michael Bogenschutz, Randi Bilberg, Angelina Isabella Mellentin, Kjeld Andersen

**Affiliations:** ^1^ Unit for Clinical Alcohol Research, Research Unit of Psychiatry, Department of Clinical Research University of Southern Denmark Odense Denmark; ^2^ Department of Psychiatry Odense Mental Health Services Region of Southern Denmark Odense Denmark; ^3^ OPEN, Open Patient Data Explorative Network Odense University Hospital Odense Denmark; ^4^ BRIDGE, Brain Research – Inter‐Disciplinary Guided Excellence University of Southern Denmark Odense Denmark; ^5^ Center on Alcohol, Substance Use, and Addictions University of New Mexico Albuquerque New Mexico USA; ^6^ Health Sciences Center The University of New Mexico Albuquerque New Mexico USA; ^7^ NYU Grossman School of Medicine New York New York USA; ^8^ Center for Digital Psychiatry Region of Southern Denmark Odense Denmark

**Keywords:** alcohol abstinence self‐efficacy scale, alcohol treatment outcomes, alcohol use disorder, latent profile analysis, older adults, relief craving, reward craving

## Abstract

**Background:**

Craving alcohol for reward (positive reinforcement) and relief (negative reinforcement) has been proposed as useful phenotypes for precision medicine approaches to alcohol use disorder (AUD) treatment. This study examined reward and relief craving in nonpharmacological treatments, Motivational Enhancement Therapy (MET) versus MET + Community Reinforcement Approach (CRA), among older adults.

**Methods:**

Secondary analyses of data from The Elderly Study (*N* = 693; mean age 64.0 years; male 59.7%), a single‐blinded, multisite, randomized controlled trial of two nonpharmacological treatments in an elderly population (60+ years) diagnosed with DSM‐5 AUD. Latent profile analysis (LPA) was used to identify craving profiles based on The Alcohol Abstinence Self‐Efficacy Scale (AASE) temptation subscale scores. The classification performance of clinical cutoff scores on the AASE scale was tested against the LPA solution. Associations between cutoff‐based craving groups and treatment success (binary variable representing change in alcohol consumption and quality of life across profiles pre‐/posttreatment) were analyzed using logistic regression, stratified on MET versus MET + CRA. Differences in alcohol consumption and quality of life scores pre‐/posttreatment were analyzed using the Wilcoxon signed‐rank test.

**Results:**

Four reward‐relief craving profiles were identified but were more distinguished by variation in relief craving (low relief, medium‐low relief, medium‐high relief, and high relief). Compared to the low relief craving group, the medium‐high relief craving group had lower odds for treatment success when receiving MET: adjusted Odds Ratio (aOR) 0.42 (95% CI 0.21–0.84), and the high relief craving group had lower odds for treatment success when receiving MET + CRA: aOR 0.38 (95% CI 0.15–0.94). Alcohol consumption was reduced, and psychological quality of life was improved at follow‐up across all relief craving groups.

**Conclusion:**

This study identified reward and relief drinking craving among older adults with AUD. Results indicate that considering relief craving when offering nonpharmacological treatment to older adults suffering from AUD may be clinically relevant.

## Graphical abstract

Relief craving, more than reward craving, defined four distinct profiles among older adults with alcohol use disorder in this secondary analysis of an RCT. Individuals with higher relief craving were less likely to benefit from nonpharmacological treatments. These findings highlight the importance of considering relief craving in tailoring interventions for older adults with AUD, supporting precision approaches in clinical addiction treatment.

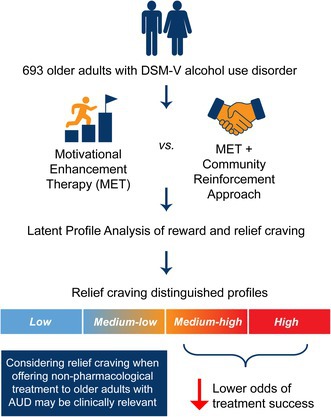

## INTRODUCTION

Craving is one of the DSM‐5 diagnostic criteria for alcohol use disorder (AUD) and is common among patients with AUD (American Psychiatric Association, 2013; World Health Organization, 2018; Yoon et al., 2006). Craving symptoms have been further characterized as either reward or relief craving. Reward craving relates to the desire to achieve positive mood states or social reward via drinking, and has been found to be associated with the percentage of drinkers in the individual's social network (Roos et al., 2017), lower drinking refusal self‐efficacy in social settings (Hasking et al., 2015), and positive expectations of the effects of alcohol (Harnett et al., 2013). In contrast, relief craving arises from states of negative mood and withdrawal sensations, resulting in the consumption of alcohol to reduce these symptoms (Koob, 2003, 2013; Koob & Volkow, 2010). Relief craving is found to be associated with more severe alcohol‐related problems (Catanzaro & Laurent, 2004; Cooper et al., 1995; Grant et al., 2007; McNally et al., 2003), higher dependence severity, and depressive symptoms (Roos et al., 2017). The individual may have primarily reward or relief drinking tendencies, and thus, subgroups of individuals are reported to have different craving profiles (Glockner‐Rist et al., 2013).

Pharmacological treatment outcomes across reward‐relief craving profiles have been studied and seem to indicate that naltrexone may work particularly well for individuals with greater reward craving, and acamprosate may be more effective for individuals with greater relief craving (Mann et al., 2018; Votaw et al., 2022; Witkiewitz et al., 2019). Whether craving profiles modify nonpharmacological treatment outcomes is sparsely investigated and of particular interest since nonpharmacological treatments are more commonly used than pharmacological agents in the treatment of AUD (Mason & Heyser, 2021).

Motivational Enhancement Therapy (MET) represents a standard nonpharmacological treatment approach for AUD that aims to enhance patients' intrinsic motivation to reduce drinking (Miller, 1992). MET employs empathetic reflections and highlights discrepancies between current behavior and personal goals to elicit motivation for change. The approach includes identification of drinking patterns, risk situations, and potential change strategies. MET can be implemented as a brief and short stand‐alone intervention but can also be combined with other treatment approaches such as the Community Reinforcement Approach (CRA) (Miller et al., 1999). CRA, based on operant conditioning principles, focuses on achieving and supporting abstinence by incentivizing sobriety and increasing positive environmental reinforcement. This approach emphasizes recreational activities and mobilization of community, family, and social networks to support change. The combination of MET and CRA provide a longer lasting and more comprehensive treatment approach and takes the patients' environment into account during treatment (Miller et al., 1999). Little is known about how different profiles of craving tendencies relate to the outcomes of these commonly delivered nonpharmacological treatment options. For example, it could be hypothesized that individuals with greater reward craving may benefit more from CRA than MET, given that CRA focuses on increasing access to alcohol‐free rewards and provides structured support for implementing these strategies in a real‐world setting.

If individuals with specific craving profiles experience better outcomes from different treatment interventions, it opens the possibility of matching specific interventions to specific individuals based on the craving profiles. However, this is only practical in daily clinical practice if it is possible for the clinician to identify the individual patient's craving profile easily and reliably.

Craving dimensions have been operationalized into questionnaires to identify individuals based on their craving patterns (Ooteman et al., 2006, Martinotti et al., 2013, Adams et al., 2016, DiClemente et al., 1994, Davis et al., 1987), such as the Alcohol Abstinence Self‐Efficacy scale (AASE) Temptation subscale (DiClemente et al., 1994). Although the AASE temptation items do not directly assess craving experiences, the temptation to drink in negative and positive settings has been used as a proxy for relief and reward craving (Glockner‐Rist et al., 2013) premised on the conceptualization of craving as a three‐pathway psychobiological model (Verheul et al., 1999). The AASE asks about specific emotional states and situations that could trigger a desire to drink, and the degree of these context‐specific urges and behavioral tendencies to respond to certain cues has thus been used as proxies to identify reward‐relief craving in a treatment‐seeking AUD population (Glockner‐Rist et al., 2013). By utilizing the AASE items as proxies for craving, Glöckner‐Rist et al. identified four separate craving groups: an overall high craving group, a high reward craving group, a high relief craving group, and an overall low craving group (Glockner‐Rist et al., 2013). While continuous scores of cravings offer valuable insights into the spectrum of craving intensity, categorizing these scores using clear cutoff points provides several advantages for clinical practice. It may enable the identification of distinct craving profiles, each with unique clinical implications (Roos et al., 2017) and the approach simplifies complex data, making it more accessible and actionable for clinicians. Additionally, discrete categories such as cutoffs allow for targeted interventions tailored to specific craving subgroups, which may enhance treatment outcomes and promote integration of findings into real‐world settings (Addolorato et al., 2005, Maisto et al., 2014, Mann et al., 2018, Votaw et al., 2022).

To date, questionnaires such as the AASE have mainly been developed based on middle‐aged treatment‐seeking AUD populations (Glockner‐Rist et al., 2013; Votaw et al., 2022) and these scales, along with reward and relief craving profiles, may not be normative for older individuals with AUD, whose numbers are growing in society and AUD treatment (Emiliussen et al., 2017; United Nations Population Division, 2017). As such, identifying reward‐relief craving profiles in an older adult treatment‐seeking AUD population and investigating whether craving profiles moderate nonpharmacological treatment responses is important.

The objectives of this study were to: (1) identify reward‐relief craving profiles and clinical cutoff scores based on profiles among older adult patients using the AASE Temptation Subscale, and (2) determine whether the identified profiles moderate treatment response in two nonpharmacological AUD treatments of different duration: MET versus MET + CRA, measured as change in alcohol consumption patterns and in quality of life across the identified profiles before and after treatment.

## MATERIALS AND METHODS

This study is a secondary analysis of the data from the Elderly Study (Andersen et al., 2015). In addition to utilizing AASE to identify reward and relief profiles, analyses of the profiles' moderation of treatment response were based on the definition of treatment response in the Elderly Study using the same covariates as in the Elderly Study.

### The Elderly Study

#### Design

The Elderly Study was an international, multicenter, single‐blinded, randomized controlled trial with 1:1 allocation conducted at six sites in three countries: Denmark (Odense, Aarhus, and Copenhagen), Germany (Munich and Dresden), and the United States (Albuquerque, New Mexico). The Elderly Study investigated possible benefits of adding the CRA (Miller et al., 1999) adapted for Seniors (CRA‐S) (Andersen et al., 2015; Moyers et al., 2013) to MET (Miller, 1995). Patient assessments were conducted at baseline and Weeks 4, 12, 26, and 52. The MET treatment focused on motivation enhancement and planning toward stopping or reducing alcohol consumption. The CRA utilized familial, recreational, social, and vocational reinforcement in AUD treatment (Andersen et al., 2015). A “Senior” module, specifically developed for the CRA used in the Elderly Study, addressed aspects of becoming older, for example, loss of physical and mental abilities. All participants were offered four sessions of MET. Participants randomized to the extended treatment condition (MET + CRA‐S) were offered up to eight additional CRA‐S sessions, totaling up to 12 sessions. The Elderly Study found no difference in treatment success defined as a Blood Alcohol Content (BAC) ≤0.5‰ at all times during the 30 days leading up to the 26‐week follow‐up time point between MET and MET + CRA (Andersen et al., 2020).

#### Sample

The Elderly Study participants were recruited from 2014 to 2016, based on self‐referrals and referrals from general practitioners and hospitals located in Denmark (three sites), Germany (10 sites), and the United States (one site). The full sample included 693 participants: MET (*n* = 351), MET and CRA (*n* = 342). All participants received oral and written information on the study and signed a written informed consent form prior to enrollment.

#### Inclusion criteria

Participants were included in the study if they: (1) were ≥60 years of age at the time of screening; (2) had a past 12‐month DSM‐5 diagnosis of AUD (American Psychiatric Association, 2013); and (3) passed a consent comprehension quiz after consent (≥8 of 10 correct answers) to ensure adequate comprehension of the study.

#### Exclusion criteria

At time of screening, participants were excluded if they had: (1) current symptoms of psychosis; (2) severe depression; (3) diagnosis of bipolar disorder; (4) suicidal thoughts or behavior; (5) use of unprescribed opioids and/or stimulants; (6) participation in other alcohol treatment programs within the last 30 days, not counting detoxification without further treatment; (7) legally appointed representatives.

#### Measures

##### Alcohol use

Alcohol use was registered using The Form‐90 questionnaire (Miller and Del Boca, 1994), a structured interview on alcohol consumption in a time line follow‐back format at baseline and at all follow‐up time points.

#### Drinking temptation and dependence severity

Temptation to drink alcohol and self‐efficacy to abstain from drinking was measured by means of The Alcohol Abstinence Self‐Efficacy (AASE) Scale (DiClemente et al., 1994) at baseline and all follow‐up time points. AASE is a 40‐item questionnaire measuring the current level of temptation to drink alcohol and self‐efficacy to abstain from drinking. The scale applies 20 high‐risk situations representing typical drinking cues. Twenty items pertain to temptation levels, and the other 20 items to self‐efficacy and self‐confidence in one's ability to abstain from drinking. Among the 20 items pertaining to temptation across risk situations, five items have been found to correlate to reward craving and five items have been found to correlate to relief craving in three large study populations (Glockner‐Rist et al., 2013; Roos et al., 2017). The items of the AASE scale reflecting relief temptation are Items 3, 6, 12, 16, and 18, while items reflecting reward temptation are 4, 8, 15, 17, 20 (Glockner‐Rist et al., 2013; Roos et al., 2017). Each item is scored on a 1–5 Likert‐type scale: 1 (not tempted at all) to 5 (extremely tempted) and ranges from 5 to 25 points.

The severity of the alcohol dependence was measured by means of The Alcohol Dependence Scale (ADS) (Skinner & Horn, 1984) at baseline and at follow‐ups Weeks 26 and 52. AUD severity was categorized based on the four quartiles of the sum‐score of the ADS: none/mild (1st quartile), moderate (2nd quartile), and severe (3rd and 4th quartiles).

#### Mental health and quality of life

The Mini International Neuropsychiatric Interview 5.0.0 (M.I.N.I.) (Hergueta et al., 1998) was used to assess AUD, mood, and anxiety disorders. The Drinker Inventory of Consequences version 2R for recent consequences (DRINC‐2R) (Miller et al., 1995) was used to measure six domains of consequences related to alcohol consumption within the past 3 months. The WHO Quality of Life‐BREF questionnaire (WHOQOL‐BREF) (Skevington et al., 2004) was used to assess subjective quality of life in four domains: physical health, psychological health, social relationships, and environment. The WHOQOL‐BREF questionnaire is validity and reliability tested among 60–95‐year‐olds in Canada and Norway (Kalfoss et al., 2008; Skevington et al., 2004). The M.I.N.I., DRINC‐2R, and the WHOQOL‐BREF were all applied at baseline and at all four follow‐up time points.

#### Demographics

Data on country, gender, age, cohabitant status, educational level, and previously received treatment for AUD were also collected.

#### Outcome variables

##### Primary outcome

Treatment success was defined as a blood alcohol content (BAC) ≤0.5‰ at all times during the 30 days leading up to the 26‐week follow‐up time point. BAC was calculated by the Form‐90 questionnaire using the Widmark formula (Posey & Mozayani, 2007; Searle, 2015)

##### Secondary outcomes

The secondary outcomes comprised mean scores of the quality‐of‐life domain scores from the WHOQOL‐BREF at 26 weeks of follow‐up, as well as various alcohol consumption measures calculated as means across 30 days at 30–0 days prior to baseline and at three and 6‐month follow‐up. The quality‐of‐life domain scores from the WHOQOL‐BREF denote a higher quality of life at higher domain scores. Mean scores in each domain were multiplied by four according to the WHO manual for scoring the questionnaire. Each domain ranges from 4 to 20 points after conversion.

The alcohol consumption measures considered were number of drinking days; number of drinks per drinking day; number of heavy drinking days defined as 4+ drinks for females/5+ drinks for males, on the same occasion; and alcohol consumption in grams/day. One standard drink was defined as 12 grams of pure alcohol.

#### Data storage

Data from the Elderly Study were stored in the Research Electronic Data Capture (REDCap) database (Harris et al., 2009), provided by Odense Patient Data Explorative Network (OPEN) (Region of Southern Denmark, 2023).

### Present study

#### Sample

**FIGURE 1:**
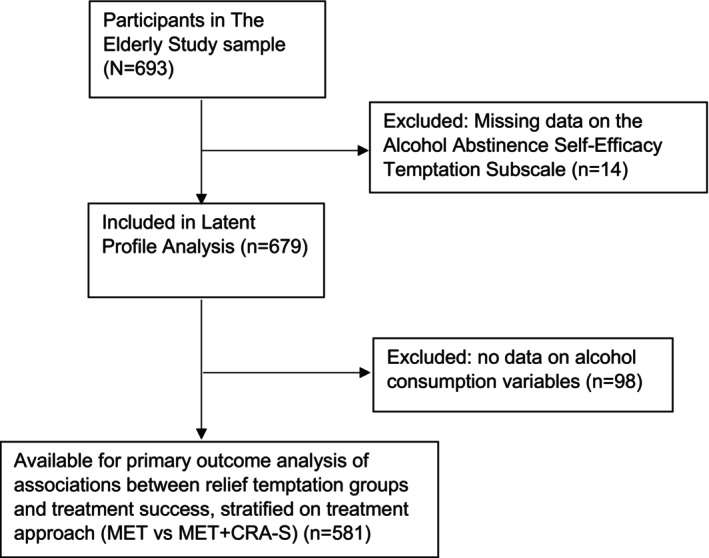
Flowchart of participants eligible for analyses. CRA‐S, community reinforcement approach senior; MET, motivational enhancement therapy.

This study was based on the 693 patients enrolled in the Elderly Study. Fourteen patients who did not complete the AASE questionnaire at baseline were excluded from the Latent Profile Analysis (LPA); see Figure 1 for details.

#### Identification of latent craving profiles

LPA (Oberski, 2016) was conducted to identify reward and relief craving profiles among the 679 patients in the Elderly Study who completed the AASE questionnaire at baseline. We utilized the same 10 AASE items previously employed as indicators for reward and relief temptation in earlier studies (Glockner‐Rist et al., 2013; Roos et al., 2017). Although the AASE does not directly measure craving, prior research has supported the use of temptation items as clinically relevant proxies for craving, as the contextual and behavioral nature of temptation seems to have the potential to differentiate individuals based on their susceptibility to specific craving mechanisms (Glockner‐Rist et al., 2013; Roos et al., 2017) (Table S1). LPAs were conducted on the same 10 reward and relief temptation items utilized in prior studies as proxies for craving; relief temptation items included Items 3, 6, 12, 16, and 18, while reward temptation items included Items 4, 8, 15, 17, and 20 (ibid). LPA tested models with 2–6 profiles, and each model was tested five times with five random seeds to assess solution stability. Each LPA was computed using random profile assignment with 100 random draws and 100 expectation–maximization (EM) iterations. Robust estimation (McLachlan et al., 2019) with robust standard error was employed due to left truncation of the reward and relief temptation scores. Interitem correlations for the 10 AASE items are provided in Table S2. The participants were assigned to the profile to which they had the highest posterior probability.

All resulting LPA profiles were stable across all five seed numbers. To determine the optimal number of profiles, we compared the LPA classifications of 2–6 profiles according to Akaike information criterion (AIC) (Sakamoto et al., 1986), Bayesian information criteria (BIC) (Schwarz, 1978), sample size adjusted BIC, entropy, and Lo–Mendell–Rubin likelihood ratio test (LMR‐LRT). We also conducted sensitivity analysis with cluster‐robust standard errors to account for potential clustering per site. The four‐profile solution was selected based on a balance of statistical indicators, clinical interpretability, consistency with prior research on craving typologies (Glockner‐Rist et al., 2013, Roos et al., 2017), and practical considerations regarding group sizes. Solutions with more than four profiles produced groups with decreasing entropy and insufficient sample sizes for meaningful analysis, while solutions with 2–3 profiles failed to capture clinically relevant distinctions in craving patterns. “Developing craving profile groups.”

We calculated the mean reward and relief score among all participants and analyzed the correlation between reward and relief scores using Pearsons's correlation coefficient. We further compared reward and relief score median and interquartile range in the four LPA profiles using ANOVA. Based on observed total item scores in the LPA profiles, we sought clinical cutoffs that were an acceptable compromise between simplification for clinical use and the detailed information found in the LPA solution. Total and mean reward and relief items scores were calculated for the best latent profile solution (described below). Given greater separation in relief scores by latent profiles (as described below), we used the relief scores to create the clinical cutoffs, called “relief craving groups.” We analyzed the classification performance of the clinical cutoffs to the LPA four‐profile solution in terms of agreement, Cohen's Kappa, sensitivity, specificity, and area under the curve (AUC).

#### Comparison of relief craving groups

Using the clinical cutoffs for the relief craving groups, we next compared the groups according to age, gender, country (site), educational level, cohabitant status, age at onset of AUD, number of previous treatment episodes for AUD, M.I.N.I. scores for anxiety and depression, DRINC‐2R scores, alcohol dependence based on ADS quartiles, alcohol consumption (grams/day; grams/drinking day; number of drinking days; number of heavy drinking days), and WHOQOL‐BREF domain scores. Associations between the relief craving groups and the covariates were analyzed using linear regression for continuous covariates, logistic regression for binary covariates, and multinomial logistic regression for categorical covariates. Pairwise comparisons of the groups were corrected for multiple testing using Šidák's correction (Abdi, 2007).

#### Associations between relief craving groups and treatment success

Associations between relief craving groups and treatment success were analyzed using logistic regression models, stratified on treatment approach (MET vs. MET + CRA). The models followed The Elderly Study protocol (Andersen et al., 2015) adjusting for country (site), gender, age, cohabitant status, educational level, AUD severity, and number of previous treatment episodes. The primary outcome of treatment success using BAC was selected to facilitate direct comparison with the primary outcome measure in The Elderly Study. The utility of BAC as a treatment outcome measure is underscored by its objectivity, precision, and demonstrated sensitivity to treatment effects (Andersen et al., 2020; Tryggedsson et al., 2023). Of the 679 patients in the LPA, 98 patients had no follow‐up alcohol consumption data, leaving 581 patients for analysis of the association between craving groups and the outcome of treatment. We did not make imputations for the 98 patients, as relying solely on their AASE scores would not strengthen the precision of the analysis.

#### Alcohol consumption patterns and quality of life before and after treatment, stratified on relief craving groups

Change from baseline to follow‐up in alcohol consumption patterns and quality of life scores was analyzed using the Wilcoxon signed rank test for each relief craving group. Analyses were conducted as total effect (any treatment) and stratified on MET and MET + CRA. Analyses were conducted in STATA version 17.

## RESULTS

In the observed study sample (*n* = 679), the majority was male (60.0%), the median age was 65.5 years (Q1 = 62, Q3 = 68), the educational level was equivalent to undergraduate (53%), never received treatment for AUD (55.4%), was living with a partner (46.8%) (Table 1), and was recruited from Denmark (*n* = 341), Germany (*n* = 189), and the United States (*n* = 149).

**TABLE 1:** Participant characteristics (*N* = 679) before treatment start.

Covariate		Missing, *n*
Age		–
Median (Q1, Q3)	64 (62.0, 68.0)	
Gender		–
Female, *n* (%)	272 (40.0)	
Male, *n* (%)	407 (60.0)	
Education		5
No degree, *n* (%)	60 (8.9)	
At most undergraduate, *n* (%)	357 (53.0)	
Graduate/postgraduate, *n* (%)	257 (38.1)	
Living with partner		–
No, *n* (%)	361 (53.2)	
Yes, *n* (%)	318 (46.8)	
Alcohol dependence^a^		3
None/mild, *n* (%)	485 (71.8)	
Moderate, *n* (%)	153 (22.6)	
Severe, *n* (%)	38 (5.6)	
Age of onset of AUD		
Median (Q1, Q3) [range]	49 (32, 59) [13–83]	176
Previous treatment episodes for AUD		–
0, *n* (%)	376 (55.4)	
1–2, *n* (%)	197 (29.0)	
3+, *n* (%)	106 (15.6)	
M.I.N.I.		
Current depression^b^, *n* (%)	18 (2.7)	–
Anxiety disorder^b^, *n* (%)	37 (5.8)	35
DrInC‐2R^c^, ^d^		
Physical, median (Q1, Q3) [range]	5 (2, 9) [0–24]	15
Interpersonal, median (Q1, Q3) [range]	4 (1, 8) [0–30]	17
Intrapersonal, median (Q1, Q3) [range]	8 (4, 13) [0–24]	15
Impulse control, median (Q1, Q3) [range]	2 (1, 4) [0–27]	15
Social responsibility, median (Q1, Q3) [range]	2 (0, 5) [0–21]	17
Control scale, median (Q1, Q3) [range]	7 (5, 10) [0–15]	13
Alcohol consumption day 30–1 before baseline		
Average consumption (g/day)		
Median (Q1, Q3) [range]	52.3 (22.5, 90.0) [0–456]	9
Average consumption (g/drinking day)		
Median (Q1, Q3) [range]	90.0 (61.4, 140.0) [12–576]	70
Number of drinking days		
Median (Q1, Q3) [range]	21 (9, 30) [0–30]	9
Number of heavy drinking days		
Median (Q1, Q3) [range]	12 (2, 25) [0–30]	9
WHOQOL^d^, ^e^		
Physical domain, median (Q1, Q3) [range]	12.6 (11.4, 14.3) [6.3–18.3]	–
Psychosocial domain, median (Q1, Q3) [range]	13.3 (12.0, 14.7) [4.0–18.0]	–
Social domain, median (Q1, Q3) [range]	13.3 (12.0, 16.0) [4.0–20.0]	–
Environment domain, median (Q1, Q3) [range]	16.0 (14.5, 17.5) [6.5–20.0]	<3

*Note*: Heavy drinking day defined as ≥4/≥5 drinks on one occasion for females/males respectively. Data presented for participants with available data on reward and relief temptation subscales on the Alcohol Abstinence Self‐Efficacy scale (*N* = 679/693).

Abbreviations: AUD, alcohol use disorder; DrInC‐2R, drinker inventory of consequences of recent drinking; g/day, grams per day; M.I.N.I, mini‐international psychiatric interview; *n*, number; Q, quantile; Ref, reference; SD, standard deviation; WHOQOL, World Health Organization Quality of Life BREF.

^a^ Based on the four quartiles in the Alcohol Dependence Scale, none/mild (1st quartile), moderate (2nd quartile), severe (3rd and 4th quartiles).

^b^ Fulfills criteria of possible current disorder (not diagnostic).

^c^ Each subscale consists of a series of questions rated on a Likert scale from 1 (never) to 3 (daily or almost daily).

^d^ The sum of each domain is calculated for each participant and medians are reported in the table.

^e^ Each domain consists of a series of questions rated on a Likert scale from 1 (very poor) to 5 (very good). Missing values are reported in categories with missing data.

### Identification of latent craving profiles

**FIGURE 2:**
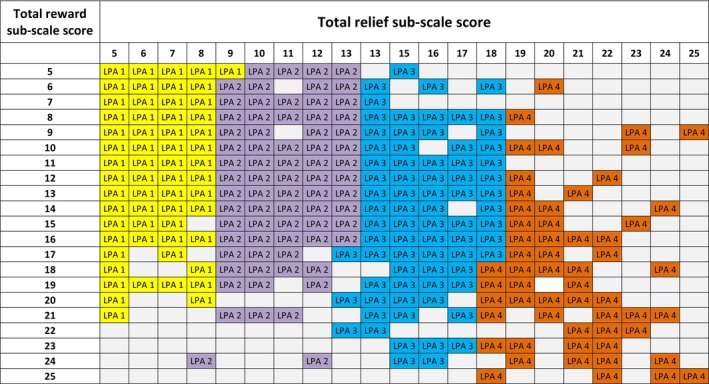
Visual representation of Latent Profile Analysis four‐profile solution according to The Alcohol Abstinence Self‐Efficacy Scale total reward and total relief subscale scores (*N* = 679). LPA, latent profile analysis. The numbers 1–4 and the color‐scheme represent the latent profile analysis four‐profile solution profiles. Total reward subscale score pertains to the total score on items number 3, 6, 12, 16, and 18 on the Alcohol Abstinence Self‐Efficacy Scale reward temptation subscale. Total relief subscale score pertains to items number 4, 8, 15, 17, and 20 on the Alcohol Abstinence Self‐Efficacy Scale relief temptation subscale. Empty cells indicate that no participant had the given combination of total relief and total reward subscale scores. As it can be seen, the four profiles are mainly categorized through total relief subscale scores (top row).

**FIGURE 3:**
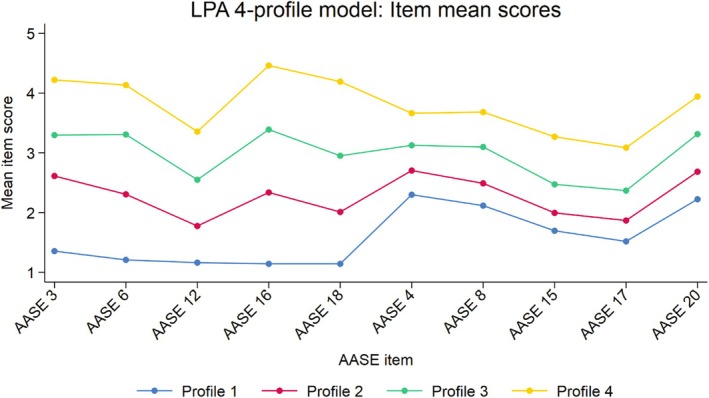
Item mean scores on The Alcohol Abstinence Self‐Efficacy Scale (AASE) reward and relief temptation items. Latent profile analysis four‐profile model (*N* = 679). LPA, latent profile analysis; AASES, Alcohol Abstinence Self‐Efficacy Scale; Relief temptation items pertain to items number 3, 6, 12, 16, and 18; Reward temptation items number 4, 8, 15, 17, and 20.

The LPA (*n* = 679) supported a four‐profile solution with high classification precision (entropy = 0.912; AIC = 8006.98; BIC = 8065.75; a‐BIC = 8109.24; LMR‐LRT = 10.91 (*p* = 0.053)) (Table 2). Results from the LPA remained unchanged when using cluster‐robust standard errors. The LPA four‐profile solution was primarily differentiated by low, medium, or high relief craving scores (Figure 2). It can be seen from Figure 2 how profiles could be characterized as: low relief (*n* = 128; 27%), medium‐low relief (*n* = 196; 29%), medium‐high relief (*n* = 104; 15%), and high relief (*n* = 197; 29%). Figure 3 presents item means scores on the AASE reward and relief temptation items for the LPA four‐profile model.

**TABLE 2:** Model fit for latent profile analysis of (*N* = 679) 60+ year old individuals with DSM‐5 diagnosed AUD observed on the Alcohol Abstinence Self‐Efficacy Scale reward and relief temptation subscales.

Model	Model fit measures					Profile sizes, *n* (%)					
	AIC	BIC	a‐BIC	Entropy	LMR‐LRT (*p*)	Profile 1	Profile 2	Profile 3	Profile 4	Profile 5	Profile 6
2‐profile	8091.242	8122.886	8146.305	0.9344	‐	287 (42.3)	392 (57.7)	N/A	N/A	N/A	N/A
3‐profile	8033.630	8078.836	8112.291	0.9238	25.11 (<0.001)	132 (19.4)	271 (39.9)	276 (40.7)	N/A	N/A	N/A
4‐profile	8006.981	8065.749	8109.240	0.9120	10.91 (0.053)	182 (26.8)	196 (28.9)	104 (15.3)	197 (29.0)	N/A	N/A
5‐profile	8000.250	8072.580	8126.108	0.8798	3.69 (0.595)	184 (27.1)	184 (27.1)	176 (25.9)	98 (14.4)	37 (5.6)	N/A
6‐profile	7982.642	8068.533	8132.099	0.8629	6.03 (0.303)	142 (29.9)	160 (23.6)	147 (21.7)	88 (13.0)	55 (8.1)	87 (12.8)

*Note*: Relief temptation subscale item pertains to items number 3, 6, 12, 16, and 18; Reward temptation subscale items number 4, 8, 15, 17, and 20 on the Alcohol Abstinence Self‐Efficacy Scale.

Abbreviations: a‐BIC, sample size adjusted BIC; AIC, akaike's information criterion; AUD, alcohol use disorder; BIC, Bayesian information criterion; DSM‐5, diagnostic and statistical manual of mental disorders, fifth edition; LMR‐LRT, Lo–Mendell–Rubin likelihood ratio test; *n*, number; *p*, *p*‐value; Profile, latent profile analysis profile.

### Developing craving profile groups

We based the clinical cutoff scores on total relief item scores only for two reasons. First, because the LPA four‐profile solution upon observation of total reward and total relief item scores could be differentiated mainly through total relief item scores (Figure 2). Second, because the correlation between the total relief and total reward item scores was moderate (*r* = 0.490).

**FIGURE 4:**
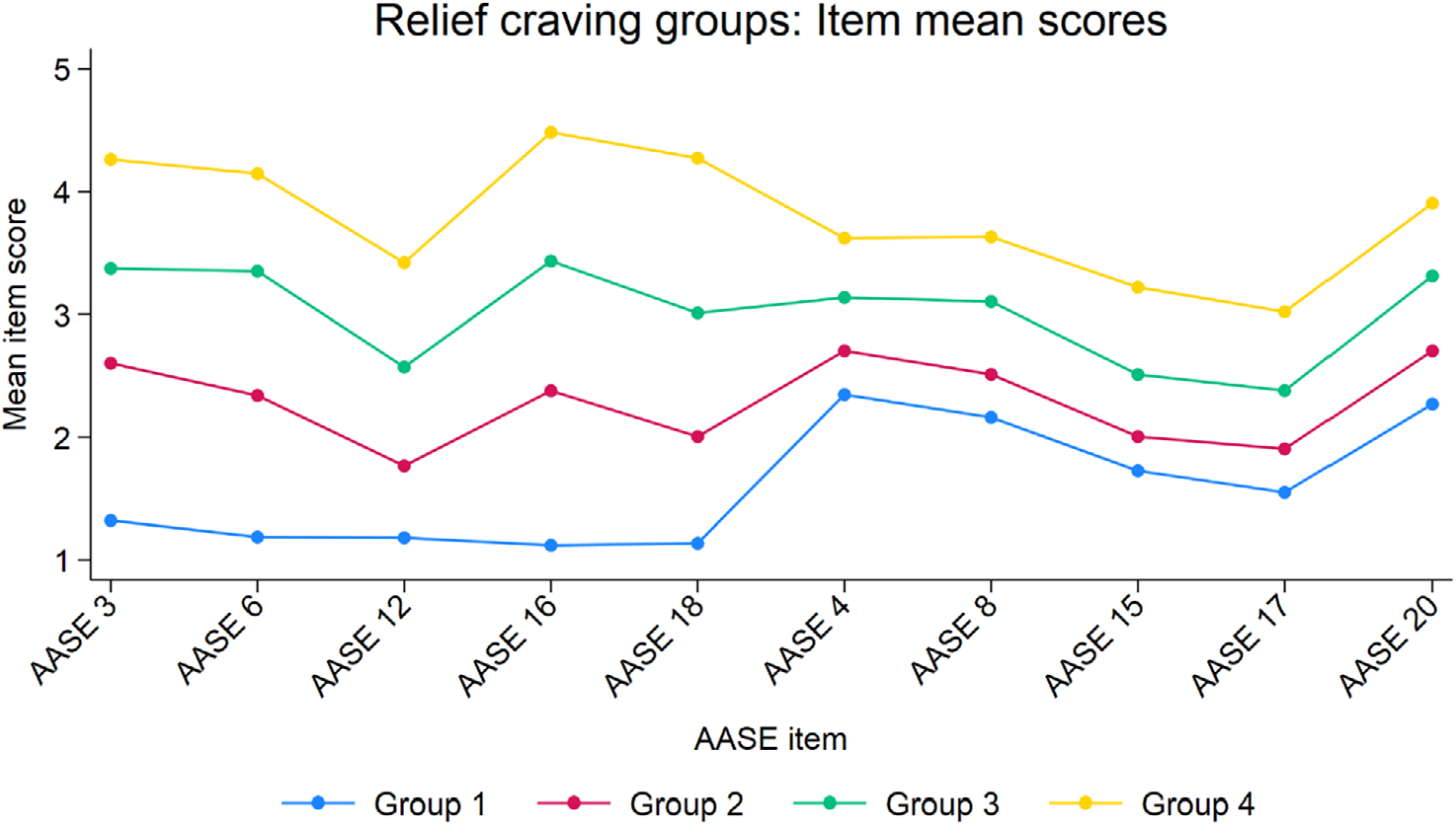
Item mean scores on The Alcohol Abstinence Self‐Efficacy Scale (AASE) reward and relief temptation items. Clinical cutoff‐based relief craving groups based on total relief subscale item scores (*N* = 679). AASE, Alcohol Abstinence Self‐Efficacy Scale; Relief temptation items pertain to AASE subscale items number 3, 6, 12, 16, and 18; Reward temptation items number 4, 8, 15, 17, and 20. Groups defined as total AASE relief subscale score <9 (low), 9–13 (medium‐low), 14–18 (medium‐high), 19+ (high).

The AASE item mean scores for the cutoff‐based relief craving groups (Figure 4) closely mirrored those of the LPA 4‐profile model (Figure 3), underscoring the similarity between the cutoff‐based groups and the LPA profiles.

The cutoff‐based craving groups, based on total relief item scores only, are denoted as *relief craving groups* henceforth.

The classification performance of the relief craving groups against the LPA four‐profile solution is presented in Table 3. The relief craving groups showed excellent classification performance: Relief craving cutoff scores <9 (low), 9–13 (medium‐low), 14–18 (medium‐high), 19+ (high) showed sensitivity (91.4–99.5), specificity (97.1–100), individual profile agreement (97.4–99.1), and AUC (95.7–98.6); and showed high overall agreement (96.5%) with Cohen's Kappa (0.95).

**TABLE 3:** Classification performance of relief craving groups against corresponding latent profile analysis four‐profile solution model (*N* = 679).

Groups	Sensitivity (%)	Specificity (%)	Agreement (%)	Area under the curve (%)
Low relief	97.5	99.8	99.1	98.6
Medium‐low relief	99.5	97.1	97.8	98.3
Medium‐high relief	95.1	98.2	97.4	96.6
High relief	91.4	100.0	98.7	95.7
Overall	–	–	96.5	–

*Note*: Relief temptation subscale score is the sum of the following items 3, 6, 12, 16, and 18 on the Alcohol Abstinence Self‐Efficacy Scale; Groups defined as relief score <9 (low), 9–13 (medium‐low), 14–18 (medium‐high), 19+ (high).

### Comparison of relief craving groups

Comparison of relief craving group characteristics at baseline is presented in Table S3. Groups of higher relief craving had a significantly greater proportion of women than lower relief craving groups (high vs. low *p* < 0.001; high vs. medium‐low *p* = 0.011; medium‐high vs. low *p* = 0.002; medium‐low vs. low *p* = 0.011). At baseline, alcohol dependence severity was higher in the high relief craving group than in the low relief craving group (*p* = 0.024). DrInC‐2R scores were overall higher in groups of higher relief (medium‐high, high), while quality of life scores were overall higher in the lower relief craving groups (medium‐low, low). Average alcohol consumption prior to treatment start was higher in the high compared to low (*p* = 0.004) and medium‐low relief craving groups (*p* = 0.001). Average consumption on drinking days was higher in the low relief craving group compared to the medium‐low relief craving group (*p* = 0.017). The number of drinking days was higher in the medium‐high group compared to the low (*p* = 0.001), and in the high compared to the low (*p* < 0.001) and medium‐low groups (*p* = 0.005). The number of heavy drinking days was higher in the high compared to low (*p* = 0.008) and medium‐low relief craving group (*p* = 0.006).

### Relief craving groups and treatment success

A total of 587 participants had complete outcome variable entries at baseline and follow‐up. With the low relief craving group as the reference group, the medium‐high relief craving group had a lower odds ratio (adjusted, aOR) for treatment success when receiving MET: aOR 0.42 (95% CI 0.21–0.84). Also, with the low relief craving group as the reference, the high relief craving group had lower odds for treatment success when receiving MET + CRA: aOR 0.38 (95% CI 0.15–0.94) (Table 4).

**TABLE 4:** Associations between relief craving groups and treatment success, stratified on treatment approach (MET vs. MET + CRA‐S). Treatment success is defined as total abstinence or controlled intake (a blood alcohol content ≤0.5‰ at all times in the 30 days before assessment). Odds ratios of treatment success at 26 weeks follow‐up with the low relief craving group as reference (*N* = 581).

	MET, *n* = 292				MET + CRA‐S, *n* = 289				Total effect (any treatment), *n* = 581			
	Treatment success, *n* (%)		OR	Adjusted OR (95% CI)	Treatment success, *n* (%)		OR	Adjusted OR (95% CI)	Treatment success, *n* (%)		OR	Adjusted OR (95% CI)
	No	Yes			No	Yes			No	Yes		
Low relief, *n* (%)	40 (46.5)	46 (53.5)	Ref.	Ref.	26 (32.5)	54 (67.5)	Ref.	Ref.	66 (39.8)	100 (60.2)	Ref.	Ref.
Medium‐low relief, *n* (%)	48 (53.3)	42 (46.7)	0.76	0.85 (0.45, 1.62)	36 (40.0)	54 (60.0)	0.72	0.78 (0.39, 1.55)	84 (46.7)	96 (53.3)	0.75	0.86 (0.55, 1.36)
Medium‐high relief, *n* (%)	53 (68.0)	25 (32.1)	0.41	**0.42 (0.21, 0.84)**	31 (40.8)	45 (59.2)	0.70	0.93 (0.44, 1.94)	84 (54.6)	70 (45.5)	0.55	0.63 (0.39, 1.02)
High relief, *n* (%)	23 (60.5)	15 (39.5)	0.57	0.70 (0.30, 1.68)	27 (62.8)	16 (37.2)	0.29	**0.38 (0.15, 0.94)**	50 (61.7)	31 (38.3)	0.41	0.55 (0.30, 1.00)

*Note*: Groups defined as relief score <9 (low), 9–13 (medium‐low), 14–18 (medium‐high), 19+ (high). Relief temptation subscale score is the sum of the following items 3, 6, 12, 16, and 18 on the Alcohol Abstinence Self‐Efficacy Scale. Logistic regression was used to compare treatment success between relief groups. Adjusted OR is adjusted for the following variables: Country (Denmark, Germany, USA), gender, age (years over 60), living with partner (yes/no), educational level (no degree, undergraduate, graduate/postgraduate), alcohol dependence severity (based on the four quartiles in the Alcohol Dependence Scale none/mild (1st quartile), moderate (2nd quartile), severe (3rd and 4th quartiles), previous treatment for AUD (0, 1–2, 3+)). Significant findings highlighted in bold.

Abbreviations: 95% CI, 95% confidence interval; CRA‐S, community reinforcement approach senior; MET, motivational enhancement therapy; OR, odds ratio; Ref., reference.

### Alcohol consumption patterns and quality of life before and after treatment, stratified on relief craving groups

Table 5 presents alcohol consumption measures and quality of life scores at baseline and at 26 weeks of follow‐up stratified on relief craving groups. All relief craving groups had lower average consumption (g/day), lower consumption on drinking days (g/drinking day), lower number of drinking days, and lower number of heavy drinking days at 26 weeks follow‐up compared to baseline.

**TABLE 5:** Alcohol consumption patterns and quality of life before and after treatment stratified on relief craving groups. Changes from baseline to 26 weeks follow‐up (*N* = 587), total treatment effect.

	Total effect any treatment											
	Group 1: Low relief (*n* = 167)			Group 2: Medium‐low relief (*n* = 183)			Group 3: Medium‐high relief (*n* = 155)			Group 4: High relief (*n* = 82)		
	Baseline	Follow‐up	*p*‐Value	Baseline	Follow‐up	*p*‐Value	Baseline	Follow‐up	*p*‐Value	Baseline	Follow‐up	*p*‐Value
Alcohol consumption day 30–1 before baseline												
Average consumption [g/day]												
Median (Q1, Q3) [range]	48.4 (18.0, 82.1) [0, 356.8]	6.0 (0, 39.6) [0, 245.4]	<0.001	50.9 (21.9, 89.4) [0, 220.4]	9.6 (0, 36.0) [0, 441.0]	<0.001	54.5 (28.8, 96.5) [0, 455.6]	20 (0, 52.6) [0, 231.0]	<0.001	80 (36.2, 103.4) [0, 444.3]	20.7 (4.6, 50.9) [0, 179.2]	<0.001
Average consumption [g/drinking day]												
Median (Q1, Q3) [range]	87.8 (60.3, 123.0) [17.8, 336.0]	56.0 (36.0, 96.0) [6.0, 245.4]	<0.001	77.6 (56.0, 136.4) [21.9, 295.2]	45.1 (31.5, 78.0) [12, 441.0]	<0.001	89.7 (54.4, 130.6) [22.5, 288.0]	57.6 (36.0, 90.0) [7.8, 241.7]	<0.001	89.4 (61.4, 142.0) [19.2, 476.0]	58.8 (39.8, 84.6) [11.8, 248.9]	<0.001
Number of drinking days												
Median (Q1, Q3) [range]	16 (5, 26) [0, 30]	3 (0, 20) [0, 30]	<0.001	20 (9, 29) [0, 30]	7 (0, 22) [0, 30]	<0.001	25 (12, 30) [0, 30]	13 (0, 28) [0, 30]	<0.001	24.5 (16, 30) [0, 30]	13 (4, 26.5) [0, 30]	<0.001
Number of heavy drinking days												
Median (Q1, Q3) [range]	10 (1, 21) [0, 30]	0 (0, 4) [0,30]	<0.001	12 (2, 22) [0, 30]	0 (0, 4) [0, 30]	<0.001	13 (2, 27) [0, 30]	0 (0, 11) [0, 30]	<0.001	16.5 (8, 28) [0, 30]	1.5 (0, 11.5) [0, 30]	<0.001
WHOQOL domains^a^												
Physical, median (Q1, Q3) [range]	13.2 (12.0, 14.3) [8.6, 18.3]	13.7 [12.6, 14.3] (5.1, 18.3)	0.145	13.1 (11.7, 14.3) [6.9, 16.6]	13.7 (12.6, 14.3) [8.6, 16.6]	<0.001	12.6 (11.4, 13.7) [6.3, 16.6]	13.7 (12.0, 14.3) [5.7, 19.4]	<0.001	12.6 (14.4, 13.7) [7.4, 17.1]	13.1 (12.0, 14.3) [8.6, 16.6]	0.001
Psychosocial, median (Q1, Q3) [range]	14.0 (12.7, 15.3) [8.0, 18.0]	14.7 (13.3, 16.0) [8.0, 18.0]	<0.001	16.7 (12.0, 14.7) [8.0, 17.3]	14 (12.7, 14.7) [7.3, 17.3]	<0.001	12.7 (11.3, 14.0) [8.0, 18.0]	13.3 (12.0, 14.7) [8.0, 18.0]	<0.001	12.7 (11.3, 14.0) [7.3, 17.3]	13.3 (12.0, 14.0) [9.3, 18.0]	<0.001
Social, median (Q1, Q3) [range]	14.7 (12.0, 16.0) [6.7, 20.0]	14.7 (12.0, 16.0) [4.0, 20.0]	0.174	14.7 (12.0, 16.0) [8.0, 20.0]	14.7 (12.0, 16.0) [5.3, 20.0]	0.271	13.3 (10.7, 14.7) [5.3, 17.3]	13.3 (10.7, 16.0) [5.3, 20.0]	0.032	12.0 (9.3, 13.3) [5.3, 20.0]	13.3 (10.7, 16.0) [6.7, 20.0]	<0.001
Environment, median (Q1, Q3) [range]	16.0 (14.5, 17.5) [11.6, 20.0]	16.5 (15.0, 18.0) [10.5, 20.0]	0.044	16.0 (14.5, 17.5) [9.5, 20.0]	16.0 (15.0, 17.5) [10.0, 20.0]	0.310	16.0 (14.0, 17.0) [6.5, 20.0]	16.0 (14.5, 17.5) [8.0, 20.0]	0.029	16.0 (14.5, 17.5) [9.0, 20.0]	16.5 (14.7, 18.0) [10.5, 20.0]	0.025

*Note*: Follow‐up at week 26. Groups defined as relief score <9 (low), 9–13 (medium‐low), 14–18 (medium‐high), 19+ (high). Relief temptation subscale score is the sum of the following items 3, 6, 12, 16, and 18 on the Alcohol Abstinence Self‐Efficacy Scale.

Abbreviations: 95% CI, 95% confidence interval; g/day, grams per day; WHOQOL, World Health Organization Quality of Life BREF.

^a^ Each domain consists of a series of questions rated on a Likert scale from 1 (very poor) to 5 (very good). The sum of each domain is calculated for each participant, and means are reported in the table. For each relief group, change from baseline to follow‐up was analyzed using the Wilcoxon signed rank test.

Across all relief craving groups, quality of life scores at follow‐up compared to baseline were higher in the physical domain (all *p ≤* 0.001) and in the psychosocial domain (all *p* < 0.001). The social domain scores were higher at follow‐up compared to baseline in the medium‐high (*p* = 0.032) and high relief craving groups (*p* < 0.001), while environmental domain scores were marginally higher at follow‐up compared to baseline in the low relief (*p* = 0.044), medium‐high (*p* = 0.029) and high relief craving groups (*p* = 0.025) (Table 5).

Further, alcohol consumption patterns and quality of life scores at baseline and follow‐up were stratified on relief craving groups and treatment approaches (MET, MET + CRA) (Tables S4 and S5). For both treatment approaches, all four relief craving groups showed lower average consumption, lower average consumption on drinking days, lower number of drinking days, and lower number of heavy drinking days at follow‐up compared to baseline.

Baseline characteristics are compared in Table S6 for those with complete follow‐up data used in the quality‐of‐life and alcohol consumption analysis (*n* = 581), those included in the development of craving groups (*n* = 679), and those lacking any AASE data (*n* = 14).

## DISCUSSION

We aimed to identify reward and relief craving profiles among older adults with clinically meaningful cutoff scores and analyze whether reward and relief craving profiles moderated treatment outcome in nonpharmacological AUD treatment in a treatment‐seeking population aged 60+ years with AUD. We did not find distinct orthogonal profiles of combined reward and relief craving as in other studies (Roos et al., 2017; Votaw et al., 2022; Witkiewitz et al., 2017, 2019), but instead profiles based on the degree of relief craving as the identifier for the latent profiles. This may be attributable to our treatment‐seeking sample, as patients with relief craving are likely more prone to seeking treatment compared to those with reward craving, who experience positive effects from drinking and may therefore not view their drinking as problematic.

Our categorical approach to conceptualizing relief craving, as opposed to a continuous one, was chosen with a clinical setting in mind. It aligns more closely with diagnostic criteria and treatment protocols and can simplify decision making and communication among healthcare providers. This approach also enhances consistency and standardization in treatment application, making it easier to establish clear thresholds for intervention. Consequently, we chose this approach aiming to provide results that are as readily applicable in clinical settings as possible. Empirically derived cutoff scores of relief craving corresponded well to the identified latent profiles and were validated by higher relief craving scores being associated with greater dependence severity and greater alcohol consumption. Individuals with higher levels of relief craving tended to have lower odds for treatment success, and this was significantly the case in the group with the highest level of relief craving. The AASE items assess the temptation to drink in specific situations, providing insights into the contextual triggers associated with different patient profiles. This focus on situational triggers aligns well with the principles of MET + CRA, which emphasizes modifying environmental cues and developing coping strategies for high‐risk situations (Andersen et al., 2020). It further aligns well with theory on psychosocial interventions for high relief craving groups focusing on improving emotion regulation, stress reduction, and coping skills, in contrast to approaches for reward‐sensitive individuals targeting social cues (Feil & Hasking, 2009; Lyvers et al., 2012). We found that compared to the low relief craving group, the high relief craving group had lower odds for a successful treatment outcome even when receiving the extended treatment approach (MET + CRA‐S). Additionally, the medium‐high relief craving group also had worse odds of treatment success when receiving short‐term treatment (MET). The higher alcohol dependence severity and lower quality‐of‐life scores in the higher relief craving groups indicate that patients with higher craving scores may represent a more challenging clinical presentation. This supports the hypothesis that patients with higher degrees of craving may have more widespread neural dysfunction than patients with less severe craving phenotypes and may not respond as well to treatments effective for other subgroups (Koob, 2013; Witkiewitz et al., 2019). These findings may also indicate that MET and CRA‐based treatments are not sufficiently targeting the symptoms presented by patients with a high or moderately high relief craving profile, highlighting the need for further research to identify effective treatment for these complex patient subgroups. Another explanation for these findings is the limited adherence to the CRA‐S module in the Elderly Study where 37% received all eight additional CRA‐S sessions, diminishing the contrast between the groups (Andersen et al., 2020). The more complex high relief craving group might necessitate a more intensive, skills‐based intervention found in a *fully delivered* CRA‐S. If they primarily received MET due to low CRA‐S adherence, this brief, motivational intervention alone might have been insufficient to address their challenges, leading to lower success rates when assigned to MET + CRA‐S. The low differentiation between the intervention groups may also help explain the low treatment success in the medium‐high relief craving group receiving MET, although this interpretation is spurious.

Regarding quality of life, the observed associations between higher dependence severity, lower quality of life, and higher craving scores are consistent with broader findings indicating that reduced drinking is associated with meaningful improvements in patients' quality of life and overall functioning, particularly for those with more severe dependence (Witkiewitz et al., 2020). Thus, successfully addressing cravings in treatment could likely lead to improvements in both physical and mental health along with social relationships, as seen in older adults treated for AUD (Tryggedsson et al., 2023).

If replicated, these findings suggest that older individuals with AUD who present to treatment with higher levels of relief craving may require different, more extensive, or simply more than nonpharmacological treatment. Future research should therefore examine, for example, whether a combination treatment of pharmacological intervention and nonpharmacological treatment would increase the probability of success, or if other nonpharmacological treatment modalities might be more effective for older treatment‐seeking individuals with high relief craving scores.

### Limitations and strengths

Several limitations should be mentioned. First, the sample was referred by a general practitioner or self‐referred to AUD treatment and had a DSM‐5 diagnosis of AUD, and the results cannot be generalized to a nontreatment‐seeking population and/or a population without an AUD diagnosis. Second, obsessive craving has been presented as a third possible craving phenotype in addition to reward and relief craving in a population of patients with alcohol dependence (Martinotti et al., 2013). This third craving dimension could not be analyzed within the present study population, as the overall study did not include instruments designed to capture this, something that is a recurring consideration for studies on craving profiles in re‐analyses on large study populations (Glockner‐Rist et al., 2013; Mann et al., 2018; Roos et al., 2017). Third, the LPA did not include the full study sample as 14 participants had no data on the AASE subscales. Analysis of treatment success included complete cases only, which resulted in a smaller sample size (*n* = 581). We selected this approach to allow a more straightforward comparison between baseline and follow‐up values, as the complete case analysis ensures the same patients are included at both time points. Imputing data for the excluded patients would not enhance the validity of the analysis since no alcohol consumption data were available for them. Future studies examining treatment retention specifically might benefit from more complex data handling approaches. It should also be noted that analyses were stratified on different treatment approaches, making for smaller groups, and therefore, the secondary outcome measures should be interpreted with caution.

The primary outcome measure of BAC reflects short‐term drinking behavior and does not encompass psychosocial aspects of recovery, such as craving reduction. Additionally, there is potential for manipulation as patients may adjust their drinking patterns prior to assessments to achieve favorable BAC results (Mann, 2004). Finally, reliance on BAC alone may overlook variations in other significant drinking patterns over time (Tryggedsson et al., 2023).

Latent profile analysis is an exploratory technique, and the results should be replicated in a new sample. The current findings of four profiles defined by low, medium‐low, medium‐high, and high reward and relief craving scores differ from prior studies that have generally found a high relief‐low reward profile and a low relief‐high reward profile. It could be the case that older adults who have longer drinking histories do not tend toward only craving for relief or only craving for reward. Also, in the current study, there was more variability in relief craving than in the reward craving items. We did not include total reward item scores in the development of clinical cutoff groups, as the correlation between reward and relief item scores was moderate, and we aimed for a simple yet clinically meaningful cutoff.

Finally, we utilized the AASE temptation items as proxies for craving. The AASE focuses on situational urges and temptations to drink, which reflect behavioral responses triggered by context or emotional states. However, “craving” is a broader concept that also encompasses a range of psychological and physiological responses related to the desire to drink (Heinz et al., 2003; Kozlowski & Wilkinson, 1987). For example, a patient may experience craving without being strongly tempted to drink in the specific situation presented by the AASE, or conversely feel tempted to drink in a particular situation without experiencing intense craving. Such discrepancies could lead to misclassification when relying solely on AASE scores to categorize patient profiles. Further, the AASE might be more effective at identifying relief drinking than reward drinking (Roos et al., 2017), and, as a retrospective self‐reported questionnaire, it is subject to biases such as recall error and desirability bias.

The strengths of the study include its large clinical sample of older adults (60+ years) diagnosed with DSM‐5 Alcohol Use Disorder (AUD) from Europe and the United States, as well as its randomized controlled trial design with random allocation between nonpharmacological treatment approaches.

## CONCLUSIONS AND FUTURE DIRECTIONS

This study proposes clinically applicable cutoff scores on an existing validated instrument for differentiating relief craving in an older population. Importantly, patients across all four identified craving profiles demonstrated reductions in alcohol consumption and improvements in quality of life scores from pre‐ to postintervention, confirming the overall effectiveness of treatment. However, our findings indicate that while relief craving does not modify the overall odds of abstinence when comparing short‐term versus extended nonpharmacological AUD treatment in older adults aged 60+ with DSM‐5 diagnosed AUD, higher relief craving levels (moderate‐high, high) were associated with decreased odds of abstinence when stratified by treatment approach. These results suggest that it may be clinically relevant to consider relief craving profiles when offering nonpharmacological treatment approaches to older adults with AUD seeking abstinence, as higher relief craving levels may influence treatment outcomes.

Future research should examine how craving profiles potentially moderate different types of pharmacological (e.g., naltrexone, acamprosate, and disulfiram) and nonpharmacological (e.g., brief alcohol intervention, contingency management, and cognitive behavioral therapy) treatment among older adults with AUD. Further, research examining other and similar nonpharmacological interventions in other populations (e.g., other age groups, nontreatment‐seeking individuals) and with respect to other craving phenotypes (e.g., obsessive craving) and clinical instruments is needed to help move the field of individualized AUD treatment forward.

## FUNDING INFORMATION

This study is a secondary analysis of The Elderly Study, which was unconditionally funded by The Lundbeck Foundation, The University of Southern Denmark, and The Region of Southern Denmark.

## CONFLICT OF INTEREST STATEMENT

The authors declare no competing interests.

## ETHICS APPROVAL

The Elderly Study was approved by the ethical committee systems following the local rules and regulations for participating in scientific research projects in all three countries: In Denmark by The Regional Scientific Ethical Committees for Southern Denmark, project‐ID S‐2013138; Germany, project‐ID EK 389102013 (the ethics committee, Technische Universitäet, Dresden) and Ethical Board of the German Society of Psychology (DGPs, Reg.‐No. EK‐ Antrag Pfeiffer‐Gerschel/Bühringer 12/2013, Munich); and the USA, New Mexico, project‐ID University of New Mexico HRRC #13‐580.

## Supporting information


Table S1

Table S2

Table S3

Table S4

Table S5

Table S6

## Data Availability

Data sharing not applicable—no new data generated.
