# Supplementary material for: Relief craving severity moderates nonpharmacological treatment outcomes in treatment‐seeking older adults with alcohol use disorder
Source: Alcohol Clin Exp Res (Hoboken). 2025 Jun 18;49(8):1803–17. doi: 10.1111/acer.70097 (PMC12365585; doi:10.1111/acer.70097)
Supplement: Supplementary file 1 — Table S1 [file ACER-49-1803-s005.docx]

**Supplementary Table 1.** Items on the Alcohol Abstinence Self-Efficacy scale pertaining to relief and reward temptation.

| Item number | Situation |
| --- | --- |
| Relief temptation | |
| 3 | When I am feeling depressed |
| 6 | When I am very worried |
| 12 | When I am physically tired |
| 16 | When I sense everything is going wrong for me |
| 18 | When I am feeling angry inside |
| Reward temptation | |
| 4 | When I am on vacation and want to relax |
| 8 | When I am being offered a drink in a social situation |
| 15 | When I see others drinking at a bar or at a party |
| 17 | When people I used to drink with encourage me to drink |
| 20 | When I am excited or celebrating with others |

Notes: Items from the Alcohol Abstinence Self-Efficacy Scale by DiClemente, Montgomery, Hughes (1994). Each item is scored on a 1-5 Likert-type scale from 1 (not tempted to drink at all) to 5 (extremely tempted to drink).
