# Supplementary material for: Relief craving severity moderates nonpharmacological treatment outcomes in treatment‐seeking older adults with alcohol use disorder
Source: Alcohol Clin Exp Res (Hoboken). 2025 Jun 18;49(8):1803–17. doi: 10.1111/acer.70097 (PMC12365585; doi:10.1111/acer.70097)
Supplement: Supplementary file 2 — Table S2 [file ACER-49-1803-s002.docx]

**Supplementary Table 2**: Descriptive statistics and pairwise correlations between the ten AASE items constituting the relief and reward scales, N = 679

|  |  | AASE items in relief scale | | | | | AASE items in reward scale | | | | |
| --- | --- | --- | --- | --- | --- | --- | --- | --- | --- | --- | --- |
|  |  | Pairwise Spearman correlations | | | | | | | | | |
| **AASE items in relief scale** | Mean (SD) | 3 | 6 | 12 | 16 | 18 | 4 | 8 | 15 | 17 | 20 |
| 3 | 2.68 (1.28) | 1 |  |  |  |  |  |  |  |  |  |
| 6 | 2.54 (1.31) | 0.61 | 1 |  |  |  |  |  |  |  |  |
| 12 | 2.04 (1.19) | 0.41 | 0.42 | 1 |  |  |  |  |  |  |  |
| 16 | 2.60 (1.42) | 0.61 | 0.63 | 0.39 | 1 |  |  |  |  |  |  |
| 18 | 2.34 (1.33) | 0.54 | 0.52 | 0.46 | 0.64 | 1 |  |  |  |  |  |
| **AASE items in reward scale** |  |  |  |  |  |  |  |  |  |  |  |
| 4 | 2.85 (1.29) | 0.27 | 0.31 | 0.32 | 0.25 | 0.21 | 1 |  |  |  |  |
| 8 | 2.73 (1.39) | 0.27 | 0.31 | 0.35 | 0.28 | 0.26 | 0.40 | 1 |  |  |  |
| 15 | 2.23 (1.32) | 0.28 | 0.26 | 0.33 | 0.31 | 0.29 | 0.38 | 0.58 | 1 |  |  |
| 17 | 2.09 (1.31) | 0.22 | 0.26 | 0.36 | 0.29 | 0.34 | 0.34 | 0.49 | 0.45 | 1 |  |
| 20 | 2.91 (1.33) | 0.28 | 0.32 | 0.38 | 0.32 | 0.33 | 0.48 | 0.56 | 0.53 | 0.52 | 1 |

All pairwise Spearman correlations are strongly significant with *p* < 0.001.
