# Supplementary material for: Relief craving severity moderates nonpharmacological treatment outcomes in treatment‐seeking older adults with alcohol use disorder
Source: Alcohol Clin Exp Res (Hoboken). 2025 Jun 18;49(8):1803–17. doi: 10.1111/acer.70097 (PMC12365585; doi:10.1111/acer.70097)
Supplement: Supplementary file 3 — Table S3 [file ACER-49-1803-s004.docx]

**Supplementary Table 3**: Comparison of patient characteristics between relief craving groups (N=679).

|  | Group 1: | Group 2: | Group 3: | Group 4: | 1v2 | 1v3 | 1v4 | 2v3 | 2v4 | 3v4 |
| --- | --- | --- | --- | --- | --- | --- | --- | --- | --- | --- |
|  | Low relief (n=193) | Medium-low relief (n=209) | Medium-high relief (n=182) | High relief (n=95) | *p*-value | *p*-value | *p*-value | *p*-value | *p*-value | *p*-value |
| **Age** |  |  |  |  |  |  |  |  |  |  |
| Median (Q1, Q3) | 65 (62, 69) | 64 (62, 69) | 65 (62, 68) | 64 (62, 67) | 0.642 | 0.572 | 0.237 | >0.999 | 0.927 | 0.965 |
| **Gender** |  |  |  |  |  |  |  |  |  |  |
| Male, n (%) | 143 (74.1) | 124 (59.3) | 102 (56.0) | 38 (40.0) | 0.025 | 0.005 | <0.001 | 0.991 | 0.013 | 0.062 |
| Female, n (%) | 50 (25.9) | 85 (40.7) | 80 (44.0) | 57 (60.0) |  |  |  |  |  |  |
| **Education** |  |  |  |  |  |  |  |  |  |  |
| No degree, n (%) | 21 (10.9) | 12 (5.8) | 18 (10.0) | 9 (9.5) | 0.759 | >0.999 | 0.762 | 0.707 | 0.871 | 0.756 |
| At most undergraduate, n (%) | 104 (54.2) | 109 (52.7) | 99 (55.0) | 45 (47.4) |  |  |  |  |  |  |
| Graduate/postgraduate, n (%) | 67 (34.9) | 86 (41.5) | 63 (35.0) | 41 (43.2) |  |  |  |  |  |  |
| **Living with partner** |  |  |  |  |  |  |  |  |  |  |
| No, n (%) | 97 (50.3) | 116 (55.5) | 94 (51.7) | 54 (56.8) | 0.553 | 0.972 | 0.752 | 0.976 | >0.999 | 0.987 |
| Yes, n (%) | 96 (49.7) | 93 (44.5) | 88 (48.4) | 41 (43.2) |  |  |  |  |  |  |
| **Alcohol dependence**^a^ |  |  | - |  |  |  |  |  |  |  |
| None/mild, n (%) | 150 (78.1) | 159 (76.1) | 120 (66.3) | 56 (59.6) | 0.452 | <0.001 | 0.001 | 0.014 | 0.017 | 0.662 |
| Moderate, n (%) | 36 (18.8) | 43 (20.6) | 44 (24.3) | 30 (31.9) |  |  |  |  |  |  |
| Severe, n (%) | 6 (3.1) | 7 (3.4) | 17 (9.4) | 8 (8.5) |  |  |  |  |  |  |
| **Age at onset of AUD** |  |  |  |  |  |  |  |  |  |  |
| Median (Q1, Q3) | 47 (30, 60) | 49 (35, 59) | 50 (35, 60) | 45 (28, 58) | 0.977 | 0.986 | 0.982 | >0.999 | 0.722 | 0.750 |
| **Previous treatment episodes for AUD** |  |  |  |  |  |  |  |  |  |  |
| 0, n (%) | 108 (56.0) | 108 (51.7) | 107 (58.7) | 53 (55.8) | 0.494 | 0.647 | 0.421 | 0.951 | 0.871 | 0.934 |
| 1-2, n (%) | 55 (28.5) | 63 (30.1) | 49 (26.9) | 30 (31.6) |  |  |  |  |  |  |
| 3+, n (%) | 30 (15.5) | 38 (18.2) | 26 (14.3) | 12 (12.6) |  |  |  |  |  |  |
| **AASE** |  |  |  |  |  |  |  |  |  |  |
| Relief score, mean (SD)  *median (Q1, Q3) [range]* | 5.94 (1.12)  *5 (5, 7) [5, 8]* | 11.09 (1.48)  *11 (10, 13) [9, 13]* | 15.74 (1.36)  *16 (15, 17) [14, 18]* | 20.59 (1.61)  *20 (19, 22) [19, 25]* | <0.001 | <0.001 | <0.001 | <0.001 | <0.001 | <0.001 |
| Reward score, mean (SD)  *median (Q1, Q3) [range]* | 10.05 (4.59)  *9 (6, 13) [5, 24]* | 11.83 (4.44)  *12 (8, 15) [5, 24]* | 14.45 (4.33)  *14.5 (11, 18) [5, 25]* | 17.40 (4.33)  *18 (15, 20) [6, 25]* | <0.001 | <0.001 | <0.001 | <0.001 | <0.001 | <0.001 |
| **M.I.N.I.** |  |  |  |  |  |  |  |  |  |  |
| *Current depression^b^* |  |  |  |  |  |  |  |  |  |  |
| Yes, n (%) | 3 (1.6) | <3 | 6 (3.3) | 8 (8.4) | N/A | 0.498 | 0.005 | N/A | N/A | 0.168 |
| No, n (%) | 190 (98.5) | N/A | 176 (96.7) | 87 (91.6) |  |  |  |  |  |  |
| *Anxiety disorder^b^* |  |  |  |  |  |  |  |  |  |  |
| Yes, n (%) | <3 | 11 (5.5) | 13 (7.7) | 12 (13.6) | N/A | N/A | N/A | 0.991 | 0.049 | 0.850 |
| No, n (%) | N/A | 189 (94.5) | 157 (92.4) | 76 (86.4) |  |  |  |  |  |  |
| **DrInC-2R^cd^** |  |  |  |  |  |  |  |  |  |  |
| Physical, median  (Q1, Q3)  [range] | 4  (2, 8)  [0, 18] | 4  (2, 7)  [0, 19] | 6  (3, 10)  [0, 22] | 7  (4, 10)  [0, 24] | 0.980 | <0.001 | 0.001 | <0.001 | 0.004 | >0.999 |
| Interpersonal, median  (Q1, Q3)  [range] | 4  (1, 7)  [0, 18] | 4  (1, 7)  [0, 22] | 5  (2,9)  [0, 28] | 5  (1, 9)  [0, 30] | 0.251 | <0.001 | 0.016 | 0.013 | 0.577 | 0.916 |
| Intrapersonal, median  (Q1, Q3)  [range] | 6  (2, 10)  [0, 21] | 7  (3, 11)  [0, 23] | 10  (6,13)  [0, 24] | 13  (7, 17)  [0, 24] | 0.003 | <0.001 | <0.001 | <0.001 | <0.001 | 0.045 |
| Impulse control, median  (Q1, Q3)  [range] | 2  (0, 3)  [0, 18] | 2  (0, 4)  [0, 14] | 3  (1, 4)  [0, 22] | 3.5  (1, 5.5)  [0, 27] | 0.278 | <0.001 | <0.001 | 0.102 | 0.060 | 0.987 |
| Social responsibility, median  (Q1, Q3)  [range] | 1  (0, 4)  [0, 15] | 2  (0, 4)  [0, 13] | 2  (1, 6)  [0, 21] | 3  (1, 6)  [0, 20] | 0.506 | <0.001 | <0.001 | 0.015 | 0.029 | 0.999 |
| Control scale, median  (Q1, Q3)  [range] | 6  (4, 9)  [0, 14] | 7  (5, 9)  [0, 15] | 8  (6, 10)  [0, 15] | 9  (6, 11)  [0, 15] | 0.009 | <0.001 | <0.001 | 0.053 | 0.014 | 0.919 |
| **Alcohol consumption day 30-1 before baseline** |  |  |  |  |  |  |  |  |  |  |
| *Average consumption [g/day]* |  |  |  |  |  |  |  |  |  |  |
| Median  (Q1, Q3)  [range] | 49.2  (15.8, 85.6)  [0, 356.8] | 47.2  (19.8, 79.2)  [0, 220.4] | 54.5  (28.8, 94.5)  [0,455.6] | 68.8  (37.1, 105.6)  [0, 444.3] | >0.999 | 0.932 | 0.014 | 0.769 | 0.005 | 0.114 |
| *Average consumption [g/drinking day]* |  |  |  |  |  |  |  |  |  |  |
| Median  (Q1, Q3)  [range] | 94.5  (68.0, 154.3)  [12.0, 576. 0] | 87.8  (60.0, 135.4)  [18.0, 300.0] | 89.7  (56.4, 130.6)  [18.0, 506.2] | 95.3  (62.7, 146.2)  [19.2, 476.0] | 0.110 | 0.578 | 0.995 | 0.964 | 0.070 | 0.335 |
| *Number of drinking days* |  |  |  |  |  |  |  |  |  |  |
| Median  (Q1, Q3)  [range] | 17  (4, 27)  [0, 30] | 19  (8, 29)  [0, 30] | 24  (12, 30)  [0, 30] | 25  (16, 30)  [0, 30] | 0.957 | 0.090 | 0.020 | 0.448 | 0.114 | 0.910 |
| *Number of heavy drinking days* |  |  |  |  |  |  |  |  |  |  |
| Median  (Q1, Q3)  [range] | 11 (1, 22)  [0, 30] | 10 (1, 22)  [0, 30] | 12 (2, 27)  [0, 30] | 17.5 (8, 29)  [0, 30] | 0.999 | 0.992 | 0.102 | 0.881 | 0.034 | 0.292 |
| **WHOQOL^de^** |  |  |  |  |  |  |  |  |  |  |
| Physical domain, median  (Q1, Q3)  [range] | 13.1  (12.0, 14.3)  [7.4, 18.3] | 12.6  (11.4. 14.3)  [6.9, 16.6] | 12.6  (114, 13.7)  [8.0, 16.6] | 12.6  (10.9, 13.7)  [7.4, 17.1] | 0.324 | 0.001 | 0.001 | 0.181 | 0.027 | 0.970 |
| Psychosocial domain, median  (Q1, Q3)  [range] | 14.0  (12.7, 15.3)  [8.0, 18.0] | 13.3  (12.0, 14.7)  [8.0, 17.3] | 12.7  (11.3, 14.0)  [6.7, 17.3] | 12.0  (11.3, 15.3)  [4.0, 17.3] | 0.014 | <0.001 | <0.001 | 0.007 | 0.006 | 0.988 |
| Social domain, median  (Q1, Q3)  [range] | 14.7  (12.0, 16.0)  [6.7, 20.0] | 14.7  (12.0, 16.0)  [6.7, 20.0] | 13.3  (10.7, 14.7)  [5.3, 17.3] | 12.0  (9.3, 13.3)  [4.0, 20.0] | 0.588 | <0.001 | <0.001 | 0.002 | <0.001 | 0.007 |
| Environment domain, median  (Q1, Q3)  [range] | 16.0  (14.5, 17.5)  [11.0, 20.0] | 16.0  (14.5, 17.5)  [9.5, 20.0] | 15.5  (14.0, 17.0)  [6.5, 20.0] | 16.0  (14.5, 17.5)  [7.5, 20.0] | 0.869 | 0.012 | 0.056 | 0.172 | 0.360 | >0.999 |

Notes: Groups defined as relief score <9 (low), 9-13 (medium-low), 14-18 (medium-high), 19+ (high). Relief temptation subscale score is the sum of the following items 3, 6, 12, 16 and 18 on the Alcohol Abstinence Self-Efficacy Scale. AUD, Alcohol Use Disorder; n, number; Q, quartile; AASE, Alcohol Abstinence Self-Efficacy Scale; M.I.N.I, Mini-International Psychiatric Interview; DrInC-2R, Drinker Inventory of Consequences of resent drinking; WHOQOL, World Health Organization Quality of Life BREF. N/A, not applicable due to GDPR since fewer than 3 events in the relief craving group, or in at least one of the compared categories.
^a^Based on the four quartiles in the Alcohol Dependence Scale, none/mild (1st quartile), moderate (2nd quartile), severe (3rd and 4th quartiles); ^b^Fulfills criteria of possible current disorder (not diagnostic); ^c^Likert scale from 1 (never) to 3 (daily or almost daily); ^d^The sum of each domain is calculated for each participant and means are reported in the table; ^e^Each domain consist of a series of questions rated on a Likert scale from 1 (very poor) to 5 (very good). Associations between relief craving group and covariates were analyzed using linear (continuous covariates), logistic (binary covariates) or multinomial logistic (categorical covariates) regression adjusted for ethnic group (country); pairwise comparisons of relief craving groups were corrected for multiple testing using Šidák’s correction.
