# Supplementary material for: Relief craving severity moderates nonpharmacological treatment outcomes in treatment‐seeking older adults with alcohol use disorder
Source: Alcohol Clin Exp Res (Hoboken). 2025 Jun 18;49(8):1803–17. doi: 10.1111/acer.70097 (PMC12365585; doi:10.1111/acer.70097)
Supplement: Supplementary file 4 — Table S4 [file ACER-49-1803-s001.docx]

**Supplementary Table 4.** Alcohol consumption patterns and quality of life before and after treatment across relief temptation groups, among patients receiving Motivational Enhancement Therapy (MET). Changes from baseline to 26 weeks follow up (n=296).

|  | **MET** | | | | | | | | | | | | |
| --- | --- | --- | --- | --- | --- | --- | --- | --- | --- | --- | --- | --- | --- |
|  | Group 1: Low relief (n=86) | | | Group 2: Medium-low relief (n=93) | | | Group 3: Medium-high relief (n=79) | | | Group 4: High relief (n=38) | | | |
|  | Baseline | Follow up | p-value | Baseline | Follow up | p-value | Baseline | Follow up | p-value | Baseline | Follow up | p-value |  |
| **Alcohol consumption day 30-1 before baseline** |  |  |  |  |  |  |  |  |  |  |  |  |  |
| *Average consumption [g/day]* |  |  |  |  |  |  |  |  |  |  |  |  |  |
| Median  (Q1, Q3)  [range] | 47.0  (4.7, 90.0)  [0, 280.0] | 7.2  (0, 44.0)  [0, 245.4] | <0.001 | 52.4  (21.3, 80.9)  [0, 220.4] | 18.4  (2.0, 43.0)  [0, 212.8] | <0.001 | 54.4  (26.7, 106.8)  [0, 211.2] | 23.3  (5.6, 67.4)  [0, 231.0] | <0.001 | 80.5  (33.8, 122.0)  [0, 444.3] | 21.2  (7.9, 58.8)  [0, 179.2] | <0.001 |  |
| *Average consumption [g/drinking day]* |  |  |  |  |  |  |  |  |  |  |  |  |  |
| Median  (Q1, Q3)  [range] | 108.0  (53.1, 148.1)  [18.0, 336.0] | 67.5  (49.4, 113.3)  [12.0, 245.4] | 0.009 | 71.3  (54.1, 110.4)  [21.3, 240.0] | 45.0  (31.9, 78.0)  [12.0, 360.0] | <0.001 | 106.0  (56.0, 144.0)  [22.5, 288.0] | 69.6  (41.4, 100.8)  [12.6, 241.7] | <0.001 | 102.4  (61.7, 138.7)  [19.2, 476.0] | 57.8  (38.6, 90.8)  [11.8, 248.9] | <0.001 |  |
| *Number of drinking days* |  |  |  |  |  |  |  |  |  |  |  |  |  |
| Median  (Q1, Q3)  [range] | 16  (3, 26)  [0, 30] | 3.5  (0,21)  [0, 30] | <0.001 | 22  (9, 30)  [0, 30] | 10  (1, 26)  [0, 30] | <0.001 | 23  (10, 30)  [0, 30] | 17  (3, 29)  [0, 30] | <0.001 | 27  (20, 30)  [0, 30] | 12  (4, 30)  [0, 30] | 0.004 |  |
| *Number of heavy drinking days* |  |  |  |  |  |  |  |  |  |  |  |  |  |
| Median  (Q1, Q3)  [range] | 9  (0, 21)  [0, 30] | 0  (0, 8)  [0, 30] | <0.001 | 9  (0.5, 22.5)  [0, 30] | 0  (0, 5)  [0, 30] | <0.001 | 12  (1, 26)  [0, 30] | 3  (0, 19)  [0, 30] | <0.001 | 22  (5, 29)  [0, 30] | 2  (0, 12)  [0, 30] | <0.001 |  |
| **WHOQOL domains^a^** |  |  |  |  |  |  |  |  |  |  |  |  |  |
| Physical, median  (Q1, Q3)  [range] | 13.3  (12.6, 14.3)  [8.6, 18.3] | 13.7  [12.6, 14.9]  (6.9, 18.3) | 0.054 | 13.1  (12.0, 14.3)  [6.9, 16.6] | 13.7  (12.6, 14.9)  [8.6, 16.6] | 0.030 | 12.0  (11.4, 13.7)  [6.3, 15.4] | 13.4  (11.4, 14.3)  [9.1, 17.1] | 0.001 | 12.6  (12.0, 13.7)  [8.0, 16.6] | 13.1  (12.0, 14.9)  [9.1, 16.6] | 0.062 |  |
| Psychosocial, median  (Q1, Q3)  [range] | 14.0  (12.7, 15.3)  [10.0, 17.3] | 14.7  (13.3, 15.3)  [8.0, 17.3] | <0.001 | 13.3  (12.0, 14.7)  [8.7, 17.3] | 14.0  (12.7, 15.3)  [7.3, 17.3] | <0.001 | 12.7  (11.3, 14.0)  [8.7, 16.7] | 13.3  (12.0, 14.7)  [8.0, 18.0] | <0.001 | 12.7  (12.0, 14.7)  [10.0, 17.3] | 14.0  (12.7, 14.7)  [10.0, 18.0] | 0.008 |  |
| Social, median  (Q1, Q3)  [range] | 14.7  (12.0, 16.0)  [8.0, 20.0] | 14.7  (13.3, 16.0)  [8.0, 20.0] | 0.160 | 14.7  (12.0, 16.0)  [9.3, 20.0] | 14.7  (12.0, 16.0)  [5.3, 20.0] | 0.636 | 13.3  (10.7, 16.0)  [5.3, 17.3] | 13.3  (10.7, 16.0)  [6.7, 20.0] | 0.063 | 12.0  (10.7, 13.3)  [6.7, 20.0] | 14.0  (10.7, 16.0)  [8.0, 18.7] | 0.007 |  |
| Environment, median  (Q1, Q3)  [range] | 16.0  (14.5, 17.5)  [11.5, 20.0] | 16.5  (15.0, 18.0)  [11.5, 20.0] | 0.032 | 16.0  (14.5, 17.5)  [10.0, 20.0] | 16.0  (15.0, 18.0)  [10.5, 20.0] | 0.856 | 15.8  (14.0, 17.0)  [9.0, 20.0] | 16.0  (14.5, 17.5)  [8.0, 20.0] | 0.047 | 16.5  (15.5, 18.0)  [9.5, 20.0] | 17.5  (16.0, 18.5)  [13.0, 20.0] | 0.027 |  |

Notes: Follow up at week 26. 95%-CI, 95% Confidence Interval; g/day, grams per day; WHOQOL, World Health Organization Quality of Life BREF; Groups defined as relief score <9 (low), 9-13 (medium-low), 14-18 (medium-high), 19+ (high). Relief temptation subscale score is the sum of the following items 3, 6, 12, 16 and 18 on the Alcohol Abstinence Self-Efficacy Scale. ^a^Each domain consist of a series of questions rated on a Likert scale from 1 (very poor) to 5 (very good). The sum of each domain is calculated for each participant and means are reported in the table. For each relief group, change from baseline to follow-up was analyzed using Wilcoxon signed rank test.
