# Supplementary material for: Relief craving severity moderates nonpharmacological treatment outcomes in treatment‐seeking older adults with alcohol use disorder
Source: Alcohol Clin Exp Res (Hoboken). 2025 Jun 18;49(8):1803–17. doi: 10.1111/acer.70097 (PMC12365585; doi:10.1111/acer.70097)
Supplement: Supplementary file 5 — Table S5 [file ACER-49-1803-s003.docx]

**Supplementary Table 5**: Alcohol consumption patterns and quality of life before and after treatment across relief temptation groups, among patients receiving Motivational Enhancement Therapy (MET) + Community Reinforcement Approach Senior (CRA-S). Changes from baseline to 26 weeks follow up (n= 291).

|  | **MET + CRA-S** | | | | | | | | | | | |
| --- | --- | --- | --- | --- | --- | --- | --- | --- | --- | --- | --- | --- |
|  | Group 1: Low relief (n=81) | | | Group 2: Medium-low relief (n=90) | | | Group 3: Medium-high relief (n=76) | | | Group 4: High relief (n=44) | | |
|  | Baseline | Follow up | p-value | Baseline | Follow up | p-value | Baseline | Follow up | p-value | Baseline | Follow up | p-value |
| **Alcohol consumption day 30-1 before baseline** |  |  |  |  |  |  |  |  |  |  |  |  |
| *Average consumption [g/day]* |  |  |  |  |  |  |  |  |  |  |  |  |
| Median  (Q1, Q3)  [range] | 48.4  (22.0, 75.6)  [0, 356.8] | 4.5  (0, 36.6)  [0.159.4] | <0.001 | 49.2  (22.8, 96.0)  [0, 216.5] | 7.2  (0, 30.8)  [0, 441.0] | <0.001 | 55.6  (29.1, 90.8)  [0, 455.6] | 17.7  (0, 42.8)  [0, 126.0] | <0.001 | 61.6  (40.5, 89.4)  [0, 360.6] | 20.3  (2.7, 42.5)  [0, 127.2] | <0.001 |
| *Average consumption [g/drinking day]* |  |  |  |  |  |  |  |  |  |  |  |  |
| Median  (Q1, Q3)  [range] | 78.2  (63.0, 109.6)  [17.8, 312.0] | 48.4  (31.3, 76.6)  [6.0, 229.3] | <0.001 | 81.4  (60.9, 149.8)  [24.0, 295.2] | 47.6  (30.0, 82.5)  [12.0, 441.0] | <0.001 | 78.8  (52,9, 118.2)  [27.2, 252.0] | 50.5  (36.0, 68.0)  [7.8, 174.0] | <0.001 | 88.2  (60.9, 155.4)  [33.1, 360.6] | 60.0  (42.0, 84.6)  [12.0, 141.3] | <0.001 |
| *Number of drinking days* |  |  |  |  |  |  |  |  |  |  |  |  |
| Median  (Q1, Q3)  [range] | 17  (5, 26)  [0, 30] | 3  (0, 20)  [0, 30] | <0.001 | 18.5  (9, 28)  [0, 30] | 4  (0, 18)  [0, 30] | <0.001 | 25.5  (14.5, 30)  [0, 30] | 10  (0, 25.5)  [0, 30] | <0.001 | 22  (16, 30)  [0, 30] | 14  (1, 24)  [0, 30] | <0.001 |
| *Number of heavy drinking days* |  |  |  |  |  |  |  |  |  |  |  |  |
| Median  (Q1, Q3)  [range] | 12  (2, 21)  [0, 30] | 0  (0, 3)  [0, 30] | <0.001 | 12  (2, 22)  [0, 30] | 0  (0, 3)  [0, 30] | <0.001 | 13.5  (0.5, 28)  [0, 30] | 0  (0, 6.5)  [0, 30] | <0.001 | 16  (8, 24)  [0, 30] | 1  (0, 8)  [0, 30] | <0.001 |
| **WHOQOL domains^a^** |  |  |  |  |  |  |  |  |  |  |  |  |
| Physical, median  (Q1, Q3)  [range] | 13.1  (12.0, 14.3)  [8.6, 16.6] | 13.1  (12.0, 14.3)  [5.1, 17.1] | 0.919 | 13.1  (11.4, 14.3)  [8.0, 16.0] | 13.7  (12.6, 14.3)  [9.7, 16.6] | <0.001 | 12.6  (11.4, 14.3)  [8.0, 16.6] | 13.7  (12.0, 14.3)  [5.7, 19.4] | 0.090 | 12.0  (10.9, 13.7)  [7.4, 17.1] | 13.1  (12.0, 14.3)  [8.6, 16.6] | 0.005 |
| Psychosocial, median  (Q1, Q3)  [range] | 14.0  (12.7, 15.3)  [8.0, 18.0] | 14.0  (12.7, 16.0)  [10.0, 18.0] | 0.018 | 12.7  (11.3, 14.7)  [8.0, 17.3] | 14.0  (12.7, 14.7)  [8.7, 17.3] | <0.001 | 12.7  (10.7, 14.0)  [8.0, 17.3] | 13.3  (12.0, 14.0)  [8.7, 17.3] | 0.031 | 12.7  (10.7, 14.0)  [7.3, 16.0] | 13.3  (12.0, 14.0)  [9.3, 16.7] | 0.027 |
| Social, median  (Q1, Q3)  [range] | 14.7  (12.0, 16.0)  [6.7, 18.7] | 14.7  (12.0, 16.0)  [4.0, 20.0] | 0.698 | 13.3  (12, 14.7)  [8.0, 20.0] | 14.7  (13.3, 16.0)  [9.3, 18.7] | 0.041 | 13.3  (10.7, 14.7)  [5.3, 17.3] | 13.3  (10.7, 14.7)  [5.3, 20.0] | 0.262 | 10.7  (9.3, 12.0)  [5.3, 18.7] | 12.0  (9.3, 14.7)  [5.3, 20.0] | 0.001 |
| Environment, median  (Q1, Q3)  [range] | 16.0  (14.5, 17.5)  [12.0, 20.0] | 16.5  (15.0, 17.5)  [10.5, 20.0] | 0.485 | 16.0  (14.5, 17.0)  [9.5, 19.5] | 16.5  (15.0, 17.5)  [10.0, 20.0] | 0.193 | 16.0  (14.5, 17.0)  [6.5, 20.0] | 16.0  (14.5, 17.5)  [9.0, 20.0] | 0.268 | 15.5  (14.0, 16.5)  [9.0, 19.5] | 15.5  (14.0, 18.0)  [10.5, 19.5] | 0.263 |

Notes: Follow up at week 26. 95%-CI, 95% Confidence Interval; g/day, grams per day; WHOQOL, World Health Organization Quality of Life BREF; Groups defined as relief score <9 (low), 9-13 (medium-low), 14-18 (medium-high), 19+ (high). Relief temptation subscale score is the sum of the following items 3, 6, 12, 16 and 18 on the Alcohol Abstinence Self-Efficacy Scale. ^a^Each domain consist of a series of questions rated on a Likert scale from 1 (very poor) to 5 (very good). The sum of each domain is calculated for each participant and means are reported in the table. For each relief group, change from baseline to follow-up was analyzed using Wilcoxon signed rank test.
