# Supplementary material for: Relief craving severity moderates nonpharmacological treatment outcomes in treatment‐seeking older adults with alcohol use disorder
Source: Alcohol Clin Exp Res (Hoboken). 2025 Jun 18;49(8):1803–17. doi: 10.1111/acer.70097 (PMC12365585; doi:10.1111/acer.70097)
Supplement: Supplementary file 6 — Table S6 [file ACER-49-1803-s006.docx]

**Supplementary Table 6**: Population characteristics before treatment start of excluded and included participants, and of included participants with data on alcohol consumption, N = 693.

|  | Excluded from analyses,  N = 14 | Included in main analyses,  N = 679 | Included in secondary analyses  on quality of life and alcohol consumption , N = 581 |
| --- | --- | --- | --- |
| **Age** |  |  |  |
| Median (Q1, Q3) | 67 (61, 72) | 64 (62.0, 68.0) | 64 (62, 68) |
| **Gender** |  |  |  |
| Female, n (%) | 7 (50) | 272 (40) | 226 (39) |
| Male, n (%) | 7 (50) | 407 (60) | 361 (62) |
| **Education** |  |  |  |
| No degree, n (%) | 0 | 60 (9) | 55 (9) |
| At most undergraduate, n (%) | 11 (79) | 357 (53) | 298 (51) |
| Graduate/postgraduate, n (%) | 3 (21) | 257 (38) | 230 (39) |
| **Living with partner** |  |  |  |
| No, n (%) | 7 (50) | 361 (53) | 310 (53) |
| Yes, n (%) | 7 (50) | 318 (47) | 277 (47) |
| **Relief score** |  |  |  |
| Median (Q1, Q3) | - | 12 (8, 16) | 12 (8, 16) |
| **Reward score** |  |  |  |
| Median (Q1, Q3) | - | 13 (9, 17) | 13 (9, 17) |
| **Alcohol dependence^a^** |  |  |  |
| None/mild, n (%) | - | 485 (72) | 417 (71) |
| Moderate, n (%) | <3 | 153 (23) | 134 (23) |
| Severe, n (%) | - | 38 (6) | 33 (6) |
| **Age of onset of AUD** |  |  |  |
| Median (Q1, Q3) | 40 (25, 56) | 49 (32, 59) | 49 (30, 59) |
| **Previous treatment episodes for AUD** |  |  |  |
| 0, n (%) | - | 376 (55) | 327 (56) |
| 1-2, n (%) | - | 197 (29) | 173 (29) |
| 3+, n (%) | <3 | 106 (16) | 87 (15) |
| **M.I.N.I.** |  |  |  |
| Current depression^b^, n (%) | 0 | 18 (3) | 15 (3) |
| Anxiety disorder^b^, n (%) | <3 | 37 (6) | 33 (6) |
| **DrInC-2R^cd^** |  |  |  |
| Physical, median (Q1, Q3) | 4 (2, 7) | 5 (2, 9) | 5 (2, 8) |
| Interpersonal, median (Q1, Q3) | 3 (1, 4) | 4 (1, 8) | 4 (1, 8) |
| Intrapersonal, median (Q1, Q3) | 4 (4, 5.5) | 8 (4, 13) | 8 (3, 12) |
| Impulse control, median (Q1, Q3) | 1 (0, 3) | 2 (1, 4) | 2 (1, 4) |
| Social responsibility, median (Q1, Q3) | 0 (0, 0) | 2 (0, 5) | 2 (0, 4) |
| Control scale, median (Q1, Q3) | 6.5 (5, 9) | 7 (5, 10) | 8 (5, 10) |
| **Alcohol consumption day 30-1 before baseline** |  |  |  |
| *Average consumption (g/day)* |  |  |  |
| Median (Q1, Q3) | 50.5 (32.4, 109.2) | 52.3 (22.5, 90.0) | 53.0 (23.3, 91.2) |
| *Average consumption (g/drinking day)* |  |  |  |
| Median (Q1, Q3) | 63.8 (44.6, 109.2) | 90.0 (61.4, 140.0) | 90.0 (61.5, 140.2) |
| *Number of drinking days* |  |  |  |
| Median (Q1, Q3) | 30 (21, 30) | 21 (9, 30) | 21 (9, 30) |
| *Number of heavy drinking days* |  |  |  |
| Median (Q1, Q3) | 20 (1, 30) | 12 (2, 25) | 12 (2, 25) |
| **WHOQOL^de^** |  |  |  |
| Physical domain, median (Q1, Q3) | 14.3 (13.7, 15.4) | 12.6 (11.4, 14.3) | 13.1 (11.4, 14.3) |
| Psychosocial domain, median (Q1, Q3) | 14.3 (12.7, 15.3) | 13.3 (12.0, 14.7) | 13.3 (12.0, 14.7) |
| Social domain, median (Q1, Q3) | 15.0 (12.0, 16.0) | 13.3 (12.0, 16.0) | 13.3 (12.0, 16.0) |
| Environment domain, median (Q1, Q3) | 17.3 (16.5, 18.0) | 16.0 (14.5, 17.5) | 16.0 (14.5, 17.5) |

Notes: n, number; AUD, Alcohol Use Disorder; Q, quantile; M.I.N.I, Mini-International Psychiatric Interview; DrInC-2R, Drinker Inventory of Consequences of Resent Drinking; g/day, grams per day; WHOQOL, World Health Organization Quality of Life BREF; heavy drinking day defined as ≥4/≥5 drinks on one occasion for females/males respectively. ^a^Based on the four quartiles in the Alcohol Dependence Scale, none/mild (1^st^ quartile), moderate (2^nd^ quartile), severe (3^rd^ and 4^th^ quartiles); ^b^Fulfills criteria of possible current disorder (not diagnostic); ^c^Each subscale consists of a series of questions rated on a Likert scale from 1 (never) to 3 (daily or almost daily); ^d^ The sum of each domain is calculated for each participant and medians are reported in the table; ^e^Each domain consists of a series of questions rated on a Likert scale from 1 (very poor) to 5 (very good).
